# Supplementary material for: MetaRibo-Seq measures translation in microbiomes
Source: Nat Commun. 2020 Jun 29;11:3268. doi: 10.1038/s41467-020-17081-z (PMC7324362; doi:10.1038/s41467-020-17081-z)
Supplement: Supplementary file 10 — Supplementary Data 7 [file 41467_2020_17081_MOESM10_ESM.zip › File2/Confidence_High_Taxonomy/132305_out.krona.html]

Javascript must be enabled to view this page.

members
magnitude
magnitudeUnassigned
count
unassigned
taxon
rank

132305\_out

23

23
2
superkingdom

phylum
23
1239

class
186801
23

2044939
23
species

SRS011134\_contig\_number\_contig-100\_2509.264905SRS011302\_contig\_number\_contig-100\_43.147551SRS012273\_contig\_number\_32319SRS013687\_contig\_number\_14300SRS014235\_contig\_number\_12348SRS019068\_contig\_number\_34524SRS020328\_contig\_number\_28217SRS024132\_contig\_number\_4795SRS042966\_contig\_number\_14717SRS049773\_contig\_number\_28410SRS049959\_contig\_number\_contig-100\_398.183078SRS064757\_contig\_number\_12015SRS1041033\_contig\_number\_contig-100\_889.110524SRS1041039\_contig\_number\_contig-100\_41081.77773SRS104311\_contig\_number\_26209SRS104400\_contig\_number\_2261SRS104636\_contig\_number\_24455SRS142599\_contig\_number\_24220SRS143895\_contig\_number\_34425SRS144183\_contig\_number\_26352SRS147139\_contig\_number\_25188SRS147557\_contig\_number\_20562SRS148196\_contig\_number\_19020
